# X-Ray based Lung Function measurement–a sensitive technique to quantify lung function in allergic airway inflammation mouse models

**DOI:** 10.1038/srep36297

**Published:** 2016-11-02

**Authors:** C. Dullin, M. A. Markus, E. Larsson, G. Tromba, S. Hülsmann, F. Alves

**Affiliations:** 1 Institute for Diagnostic and Interventional Radiology, University Medical Center Goettingen, Germany; 2 Italian Synchrotron Light Source ‘Elettra’ Trieste, Italy; 3 Max-Plank-Institute for Experimental Medicine, Dept. of Molecular Biology of Neuronal Signals, Goettingen, Germany; 4 Clinic for Anesthesiology, University Medical Center, Goettingen, Germany; 5 Department of Hematology and Medical Oncology, University Medical Center, Goettingen, Germany

## Abstract

In mice, along with the assessment of eosinophils, lung function measurements, most commonly carried out by plethysmography, are essential to monitor the course of allergic airway inflammation, to examine therapy efficacy and to correlate animal with patient data. To date, plethysmography techniques either use intubation and/or restraining of the mice and are thus invasive, or are limited in their sensitivity. We present a novel unrestrained lung function method based on low-dose planar cinematic x-ray imaging (X-Ray Lung Function, XLF) and demonstrate its performance in monitoring OVA induced experimental allergic airway inflammation in mice and an improved assessment of the efficacy of the common treatment dexamethasone. We further show that XLF is more sensitive than unrestrained whole body plethysmography (UWBP) and that conventional broncho-alveolar lavage and histology provide only limited information of the efficacy of a treatment when compared to XLF. Our results highlight the fact that a multi-parametric imaging approach as delivered by XLF is needed to address the combined cellular, anatomical and functional effects that occur during the course of asthma and in response to therapy.

Lung diseases such as asthma, emphysema and chronic obstructive pulmonary disease (COPD) continue to be a major burden of public health and show an increasing number of incidences worldwide. Especially asthma is a heterogeneous disease which occurs in several different forms and severity^1^. This is also reflected in the strong variations in the efficacy of current treatments among different patients. Due to the complex immunological nature of asthma and the rapid change of symptoms, preclinical research with *in-vivo* experimental animal asthma models is inevitable. Mouse asthma models, such as the ovalbumin (OVA) induced acute allergic inflammation, have facilitated a better understanding of the interactions in asthma, but have also revealed that many drugs that were successfully tested in mice failed in clinical trials. This is not only related to the immunological and physiological differences between mice and humans but also to the challenges a small organism such as the mouse presents for imaging and measurement of common hallmarks of asthma^2^. Thus, histology and cell based approaches such as cell count in broncho-alveolar lavage (BAL) are largely used, but how these features relate to the impairment of lung function is not well understood. In order to address lung function in mice, plethysmography techniques are usually applied, which either require intubation or restraining of the mouse^3^, adding the influence of stress to the results, or - in the case of unrestrained whole body plethysmography (UWBP) - are limited in their predictive power due to a lack in sensitivity^4^. Improved or novel small animal lung function measurement techniques are thus fundamentally needed, above all because this parameter is the most relevant in humans. New treatments are of little use if they cannot be reliably tested for improvement of lung function. Accurate lung function data in mice, in particular in response to novel therapeutic strategies, may allow a better preclinical evaluation of drug efficacy as well as a better comparison with patient data.

Here we present a novel lung function measurement approach utilizing low dose planar cinematic x-ray imaging, which we designate “X-Ray Lung Function” [XLF]. By correlating the results with histology, BAL, synchrotron phase contrast CT and UWBP we demonstrate that XLF is easy to use *in vivo* and shows a significantly higher sensitivity than UWBP for the reliable assessment of experimental allergic airway inflammation and the efficacy of dexamethasone treatment.

## Results

### General approach of XLF

**Figure 1:**
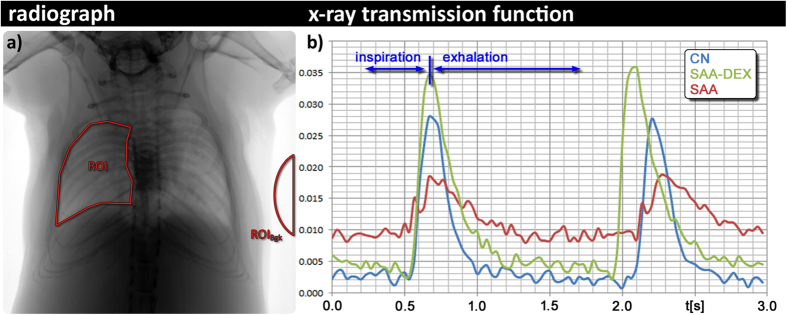
Breathing modulates the x-ray transmission at the chest region over time. (**a**) shows an exemplified radiograph: the average x-ray transmission function over time (XTF) is measured in a region of interest of the lung (ROI) and normalized by the background intensity (ROI_Bgk_); and (**b**) shows exemplary two breathing cycles of a healthy control animal CN (blue curve); a mouse from the severe acute airway inflammation model (SAA) two days after the last challenge (red curve) and a mouse from the same model that had been treated with dexamethasone before each challenging step (SAA-DEX, green curve). The SAA mouse demonstrates a strong reduction in the area under the curve due to the lower air content within the lung at peak inspiration and an asymmetric shape of the breathing event with a shorter time to peak. The treated mouse (SAA-DEX) shows XTF properties comparable to those obtained in the healthy control mouse CN.

In order to depict alterations in lung function *in-vivo*, XLF uses low dose planar cinematic radiography of the chest region of unrestrained isoflurane anesthetized mice. We performed the technique with the Quantum FX CT (Perkin Elmer), but in principal any x-ray tube with an attached camera and anesthesia could be used. The CT produces a movie of 2D radiographs capturing 34 s of the chest movements during breathing, depicting the x-ray transmission of the chest area. By slightly modifying the isoflurane concentration, the breathing frequency for each mouse was adjusted to roughly one breathing event in 1400 ms. In the generated x-ray movies, regions of interest (ROI) were placed over the left and right lung lobe at the time point of maximum contraction. For calibration purposes a region of interest was also placed in an area outside the mouse (ROI_Bkg_) (Fig. 1a). The average x-ray transmission is measured in each frame of the movie for both the ROI and ROI_Bkg_ (Supplementary Figure S1). The x-ray transmission function (XTF) over time describes the difference of these values normalized by the average background signal (ROI_Bkg_), in order to account for variations in intensity fluctuations of the x-ray tube (Supplementary Figure S1):


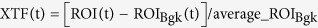


In order to account for slight drifts in the x-ray transmission function, moving average filtering (range 200) was applied and the best fitting polynomial function of an order of 2 was calculated (Poly_Bgk_) and subtracted from XTF. As the XTF represents a ratio between the x-ray transmission of the chest and the background it is without a physical unit.

Because we adjusted the breathing frequency to one breathing event in 1400 ms and we captured movies of 34 s, the XTF of each mouse contains about 21 breathing cycles (Supplementary Figure S1). Each breathing cycle was parameterized and the average of all cycles within the captured time frame was used to characterize the lung function of the mouse.

We applied the technique on an OVA induced experimental allergic airway disease mouse model mimicking severe acute asthma (SAA), consisting of 2 sensitization steps and 4 challenges. PBS sensitized and challenged mice were used as controls (CN). Treated mice received dexamethasone intraperitoneally (i.p.) 1 h before each challenge (SAA-DEX) (Supplementary Figure S2). All mice were gender and age matched and were analyzed on day 28 (before the 1^st^ OVA challenge) and on day 35 (2 days after the last challenge). Figure 1b shows two representative breathing cycles in a XTF of a SAA mouse (red), a CN mouse (blue) and a dexamethasone treated SAA mouse (green). Clear differences in the XTF-curves can be seen between SAA, DEX treated SAA and CN: XLF of the SAA mouse displayed a strong reduction in the area under the curve due to the lower air-to-soft-tissue ratio within the lung at peak inspiration and an asymmetric shape of the breathing event with a shorter time to peak. The treated mouse (SAA-DEX) showed XTF properties comparable to those obtained in the healthy control mouse CN.

### Quantitative parameters of XLF

In order to quantify the differences in the XTFs among the examined groups of mice, we parameterized the XTF curves. For this purpose we first identified breathing events within the XTF curves by calculating intersection points of the XTF curve and a line at the level of the average XTF+33% of the standard deviation of the XTF (Supplementary Figure S1). Breathing peaks were identified as intervals between two successive intersection points of this line with the XTF curve, where all values are above the level of the scan line. Each breathing peak was approximated with a third order polynom P = b_2_*(x-b_1_)^3-b_3_*(x-b_1_)^2-b_4_*(x-b_1_)+b_5_. The average of each parameter (**b**_**1**_**-b**_**5**_) over all breathing cycles is calculated and used to characterize the lung function in each particular scan. As the XTF is without physical unit, the parameters **b**_**i**_ are also unitless.

**B**_**1**_describes the shift of the position of the breathing peak maximum and is therefore a measure of the change in the ratio between inhalation and expiration time. A reduction in the relative inhalation time corresponds to an increase in **b**_**1**_. As shown in Fig. 1b, the representative XTF of a SAA mouse demonstrates an asymmetric shape of the breathing peaks with a shift of its maximum to the left and therefore an increase in **b**_**1**_. This is most likely related to the decreased elasticity of the asthmatic lung causing the common effect of expiratory airflow limitation^5^.

**B**_**2**_ and **b**_**4**_, as factors for the linear and cubic term of the polynom, both account for asymmetry in the XTF. Besides differences in the ratio between the inhalation and exhalation time, they also reflect changes in the slope of decay of the XTF in the exhalation period.

**B**_**3**_, as the factor of the quadratic term of the polynom, describes the ‘steepness’ of the breathing peak which is dramatically reduced in SAA lungs. This means that the peak is flatter due to a reduced difference in x-ray transmission between inspiration and expiration. This could be caused by two possible effects, an increased soft-tissue content in the lung and/or a reduced amount of inhaled air. **B**_**3**_ thus describes the dynamic of the gas exchange.

**B**_**5**_, as the absolute term of the polynom, represents the value of the peak maximum. The peak maximum represents the maximum air content present in the lung. A high maximum indicates more air in the lung.

**B**_**3**_, in combination with **b**_**5**_, thus describe the area under the curve, meaning how much air is transported in which time. High **b**_**3**_ and **b**_**5**_ values indicate a faster transport of a higher amount of air, as found in healthy mice. A reduction in **b**_**3**_ and **b**_**5**_ is related to lower air content and a longer exchange time and is thus related to an increase in soft-tissue due to the swelling of the airways, both hallmarks of asthma, which both lower the x-ray penetration. If **b**_**5**_ is unchanged, but **b**_**3**_ is reduced, this would mean a reduction in air flow only and could be an indication for a loss of elasticity in the absence of inflammation/oedema.

In addition, the parameter t_**in**_**[%]** was measured, indicating the time that the XTF needs to reach its maximum as percentage of the total length of a breathing event. This parameter more accurately represents the asymmetric behavior of the duration of inspiration and expiration in asthma. As shown in Fig. 1b, in the presence of allergic inflammation (SAA) the XTF is asymmetric and shows a reduced **t**_**in**_**[%].**

As detailed below, the parameters **b**_**1**,_**b**_**3**,_ **b**_**5**_ and **t**_**in**_**[%]** showed alterations of the lung function in the presence of allergic inflammation. **B**_**1**_**-b**_**4**_ were changed during bronchoconstriction as a result of exposure to methacholine.

### XLF is a reliable *in-vivo* lung function measurement method and more sensitive than UWBP

To validate the performance of XLF, three groups of mice were analyzed 2 days after the last challenge at the acute time point of the disease: healthy controls (CN, N = 6), mice from an OVA induced severe acute airway inflammation model (SAA, N = 6) and mice that received dexamethasone 1 h before each OVA challenge (SAA-DEX, N = 6).

**Figure 2:**
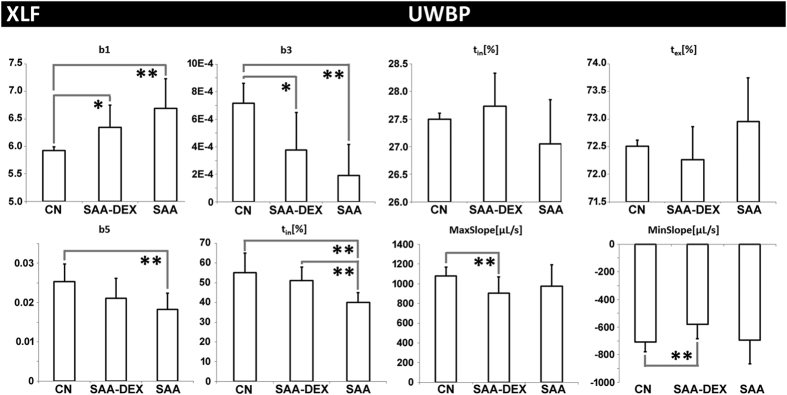
Comparison of XLF parameters with UWBP for healthy mice (CN), mice with severe acute airway inflammation (SAA) and dexamethasone treated mice of the same model (SAA-DEX). XLF shows significant differences between CN and SAA for b_1_, b_3_, b_5_ und t_in_. The treated mice (SAA-DEX) show intermediate values suggesting an inefficient treatment in terms of lung function. UWBP failed to display significant differences in all parameters between CN, SAA and SAA-DEX mice, indicating that our XLF approach - as the only other *in-vivo* LF method used in this study - has a higher sensitivity and specificity than *in-vivo* UWBP. Statistical significance difference of the results is indicated by (*p < 0.1, **p < 0.05).

XLF shows highly significant differences between CN and SAA mice (p < 0.05) in the parameters **b**_**1**_, **b**_**3**_, **b**_**5**_ and **t**_**in**_**[%]**: SAA mice are characterized by a higher **b**_**1**_ and lower **t**_**in**_**[%]**, indicating a shorter relative inspiration period. Furthermore, SAA mice display reduced **b**_**3**_ and **b**_**5**,_ pointing to decreased x-ray transmission of the lung region caused by a lower amount of air and a reduced air flow. The combination of both parameters point to reduced elasticity and increased soft tissue content and/or oedema, related to the inflammation in SAA (Fig. 2). A significantly increased **b**_**1**_ and decreased **b**_**3**_ was found in the dexamethasone treated asthmatic mice SAA-DEX when compared to the healthy controls CN, however less pronounced than in the untreated SAA mice. Interestingly, **b**_**5**_ is not significantly reduced in SAA-DEX animals when compared to CN, suggesting that the treatment has resulted in a reduction of the swelling. XLF was therefore sensitive enough to demonstrate that dexamethasone acts predominantly on the inflammatory aspects of the disease and less on the elasticity of the lung. XLF measurements directly before challenge (i.e. after sensitization) revealed no difference compared to controls in all parameters (data not shown).

UWBP reveals the same trend in **t**_**in**_**[%]** as XLF, but the differences are not significant (Fig. 2). The UWBP parameters **MaxSlope** and **MinSlope** indicate a reduction in the airflow of SAA and SAA-DEX in comparison to CN, which corresponds to the observed decrease in x-ray transmission **b**_**3**_ and **b**_**5**_ in XLF (Fig. 2). Interestingly, this effect was more pronounced in dexamethasone treated mice than in SAA mice and was significantly different to CN.

To show that XLF is able to make repeated measurements in the same animal we repeated the experiment with another cohort of mice (SAA, N = 5; CN, N = 5) and performed a second challenge after a recovery time of 19 days from the initial challenges. XLF was able to detect significant differences in parameters b_1_ and t_in_[%] between SAA and CN after rechallenge. Parameters b_3_ and b_5_ were not significantly different, suggesting that a single rechallenge led to a weaker inflammatory reaction and/or oedema as the initial 4 challenges (data not shown).

### XLF is able to measure airway responsiveness to methacholine

**Figure 3:**
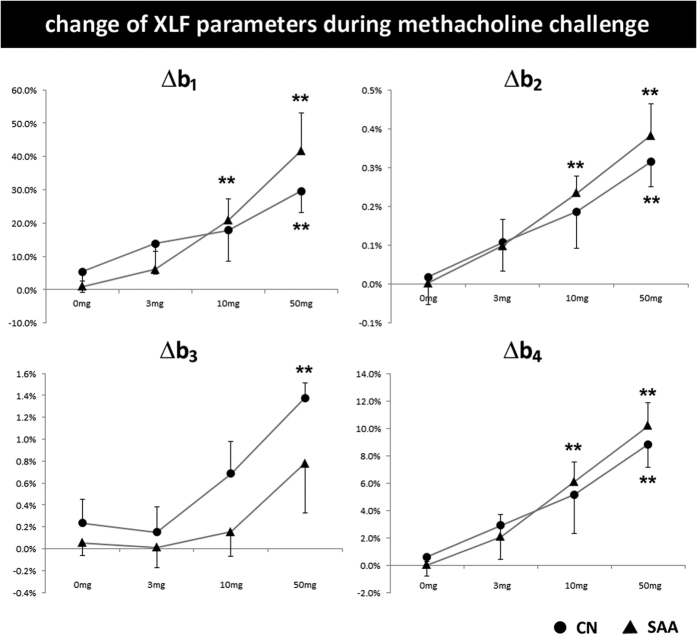
Airway responsiveness to methacholine as measured by XLF. Relative change of XLF parameters to baseline values are shown for SAA and CN in dependence of increasing concentrations of methacholine (0, 3, 10, and 50 mg/ml). In SAA animals b_1_, b_2_ and b_4_ are significantly affected by a lower concentration of methacholine (10 mg/ml) than CN (50 mg/ml) relative to 0 mg/ml (**p < 0.05).

Airway responsiveness to methacholine was determined 2 days after the last challenge. Both CN and SAA reacted in changed parameters in dependency of the concentrations of methacholine. There was higher airway reactivity in SAA mice compared with CN mice as measured by a stronger relative increase of parameters **b**_**1**_, **b**_**2**_, **b**_**3**_ and **b**_**4**_ (Fig. 3). As **b**_**2**_ and **b**_**4**_ reflect the asymmetry of the XTF curve, their change upon bronchoconstriction most likely displays the spasms caused by narrowing of the bronchi. This result shows that XLF is able to reliably measure airway responsiveness.

### XLF in comparison to BAL, histology and pSRμCT

In order to validate the findings of XLF, the results were correlated to data obtained by BAL, synchrotron phase contrast CT (pSRμCT) and histology. The parameters measured by each technique are described in more detail in Online Methods.

**Figure 4:**
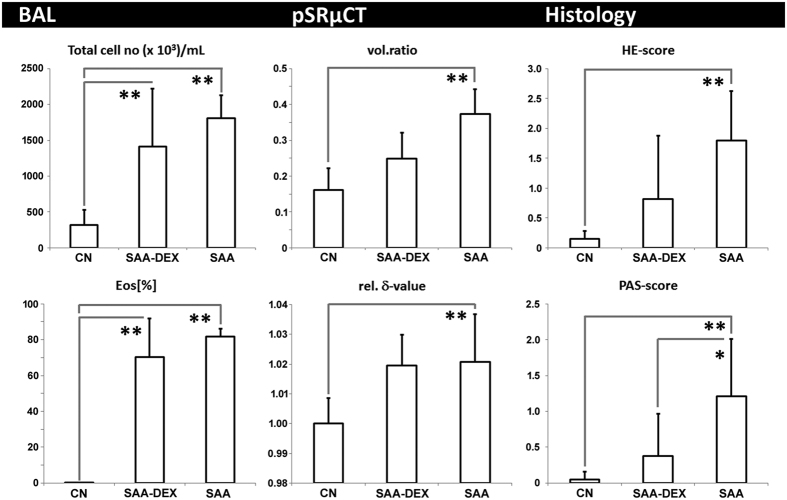
Correlation of XLF and UWBP to *ex-vivo* techniques BAL, pSRμCT, and histology for healthy mice (CN), mice with severe acute airway inflammation (SAA) and dexamethasone treated mice of the same model (SAA-DEX). The analysis of BAL shows a significant increase in total cell number and in the relative amount of eosinophils (EOS[%]) in SAA mice when compared to CN mice, verifying the presence of asthma. SAA-DEX still reveals an increased number of eosinophils suggesting inefficient treatment. pSRμCT suggests a strongly increased soft-tissue vol.ratio within the lungs of SAA and intermediate values for SAA-DEX mice. SAA and SAA-DEX lungs show no difference in the rel. δ-value. Histology shows an increased amount of infiltrating cells in SAA (HE-score) and an increased mucus production (PAS-score), SAA-DEX mice demonstrate intermediate results, suggesting only partially successful treatment. Statistical significance difference of the results is indicated by (*p < 0.1, **p < 0.05).

BAL is a common tool for diagnosis of lung inflammation and analysis of differential cell counts in BAL fluid is standard for both preclinical and clinical routine. As shown in Fig. 4, the total amount of cells in the BALs of SAA animals is significantly increased compared to controls (CN) and this increase is mostly related to an increase in eosinophils, as is characteristic for this OVA induced asthma mouse model. Moreover, the dexamethasone treated mice (SAA-DEX) showed slightly less but still a high amount of eosinophils compared to the controls (CN), indicating that based on the BAL results the dexamethasone treatment is only partly effective.

A characteristic asthma related swelling of the airways was verified by pSRμCT on *in-situ* lungs, demonstrating a significant increase in the soft-tissue **vol.ratio** of the lungs in SAA mice compared to CN (Fig. 4 and Supplementary Figure S3). These structural alterations could also be seen by histology of paraffin lung sections stained by H&E and PAS. **HE** and **PAS scores** were obtained and showed an increase in the presence of asthma, consistent with an increase in cell infiltration and mucus (Fig. 4 and Supplementary Figure S4). The dexamethasone treatment demonstrated a significantly reduced inflammation in the lungs as demonstrated by a diminished **PAS-score** of SAA-DEX in the histology and an intermediate soft-tissue **vol.ratio** of SAA-DEX between CN and SAA. These results, together with the consisting high eosinophil number in SAA-DEX mice, indicate that the allergic reaction has not completely resolved after dexamethasone treatment. This is further supported by the increased relative **δ-value** in pSRμCT of SAA-DEX mice in comparison to CN (Fig. 4). The **δ-value**, the real part of the complex refractive index, is a parameter that characterizes the composition of the lung soft-tissue^6^. A lower **δ-value** would point to a higher water content; instead, the here observed increased **δ-values** indicate an effect of an elevated amount of cells in SAA and SAA-DEX rather than mucus production. As the rel. **δ-value** is not reduced in the same way as the soft tissue **vol.ratio** for SAA-DEX lungs, this suggests that the treatment suppresses the swelling of the airway walls without affecting the composition of the tissue.

### Influence of age and sex on XLF

In order to evaluate the influence of sex and age on the XLF results, 72 female BALB/c mice divided into 3 age groups (younger than 7 weeks N = 16, between 7 and 9 weeks old N = 27 and older than 9 weeks N = 29) as well as 37 female and 32 male mice older than 7 weeks were analyzed. We found a significantly increased **b**_**5**_ and a significantly lowered **t**_**in**_**[%]** in the male compared to the female mice (Supplementary Figure S5), most likely due to the larger body size of age matched males. Additionally, **b**_**1**_, **b**_**3**_ and **b**_**5**_ show significant variations with the age of the analyzed mice (Supplementary Figure S5). The magnitudes of these variations are lower than the respective effect sizes between CN and SAA and would therefore not interfere with the detection of hallmarks of asthma. However, to minimize variability and due to previous reports on sex specific differences in the severity of asthma^7^, it is recommended that XLF be used with gender and age-matched mice, as was the case for all other experiments in this study.

## Discussion

We demonstrated that our novel lung function approach XLF, using low dose cinematic planar x-ray imaging, allows the distinction of mice with severe allergic airway inflammation (SAA) from healthy mice (CN), and the assessment of the efficacy of dexamethasone in SAA mice (SAA-DEX). We introduced quantitative parameters (**b**_**1**_–**b**_**5**_ and **t**_**in**_**[%]**) based on curve fitting of the breathing events in the x-ray attenuation over the mouse chest. SAA demonstrated a reduced **t**_**in**_**[%]**-value, indicating a shorter relative inspiration time in asthma, and a reduced **b**_**3**_**-**value depicting lower air-flow, as well as a reduced **b**_**5**_, suggesting a higher soft-tissue-to-air ratio in asthma. These results are paralleled by the strong increase in eosinophils and total cells observed in BAL and by histology. Together with the strongly increased soft-tissue **vol.ratio** and rel. **δ-value** demonstrated by SRμCT, this indicates a modification in the relation between water and cells within the lung.

Furthermore, we were able to show that XLF was sensitive enough to measure significant differences in 2 parameters of rechallenged SAA versus CN animals after 19 days of recovery from the acute inflammatory response. We could also show that XLF is capable of determining airway responsiveness to methacholine. Methacholine challenge tests are often performed by the forced oscillation technique (FOT), a very invasive and terminal method, where the mice are tracheotomized and artificially ventilated. By contrast, XLF can reliably determine airway resistance to methacholine challenge non-invasively and non-terminally. Both these findings, the ability to rechallenge over time and to measure airway resistance non-invasively, demonstrate that XLF allows longitudinal studies in the same animal. This is of immense importance as it dramatically reduces the numbers of animals needed and increases the predictive value of the results due to the absence of inter- subject variations.

Analysis of the influence of age and sex of the mice on XLF indicated significant differences for both factors, underlying the sensitivity of XLF. While these differences were much lower than those observed between SAA and CN, age and gender effects on XLF can be avoided by using age and gender matched mice.

For comparison with other lung function methods, we used UWBP over restrained plethysmography, as the latter is much more invasive and places a substantial stress on the animal, which may influence lung function data^8^. Compared to XLF, UWBP struggled with the higher breathing frequency of the non-anesthetized mice and revealed only faint differences in the lung function between treated and healthy mice. **t_in_** obtained by UWBP is the only parameter that can be directly related to **t_in_** in XLF and essentially indicates the same trend (a reduction in SAA), but could not reach significance. Although UWBP is less invasive because it does not require anesthesia, it became obvious that the method is less sensitive than XLF. One potential reason is the problem of explorative behavior which is associated with higher variability of respiratory cycles. Moreover, the lower breathing frequency in the anesthetized mice compared to the non-anesthetized mice used for UWBP, may contribute to the enhanced sensitivity of XLF. In contrast to UWBP, XLF is also influenced by structural changes within the lung that occur in allergic airway inflammation. This may further explain the increased sensitivity of XLF in discriminating between CN, SAA and SAA-DEX *in-vivo* compared to UWBP.

The minor reduction of eosinophils in the dexamethasone treated mice as well as the only marginal differences (non-significant data) in morphological changes from SAA to SAA-DEX judged by histology and pSRμCT, may suggest that the dexamethasone treatment is largely inefficient. XLF, on the one hand, shows that **b_5_** is not significantly reduced when compared to controls, while **b**_**1**_ and **b**_**3**_ are. This suggests that dexamethasone resulted in a reduction of inflammation but no improvement of the elasticity of the lung. XLF thus produced additional information on the efficacy of the treatment that could not be extracted from any of the other techniques. By comparison of these readouts we conclude that for evaluating treatment efficacy it is of crucial importance to consider and assess all aspects – cellular, anatomical and functional alterations. XLF may advance such a multimodal approach as it allows a very sensitive evaluation of functional alterations in lung disease *in-vivo* over time and can therefore be used to follow the course of the disease or the response to a treatment.

In summary, we demonstrated the benefits of XLF, a novel highly sensitive *in-vivo* lung function measurement method for mouse lung disease models. Due to the combined influence of functional and morphological alterations on the measured parameters, XLF provides additional information that can help to better understand the pathomechanism of lung disease as well as the effect of novel therapies.

## Methods

### Animal models for allergic airway disease

Female BALB/c mice (4–6 weeks old) were purchased from Charles River Laboratories and maintained with ad libitum food and water. An OVA induced experimental allergic airway disease model was generated to mimic severe acute asthma (SAA) as described by Dullin *et al*.^6^. Briefly, mice were sensitized twice with 50 μg OVA i.p. and i.n. on days 0 and 14 and then challenged 4 times on days 28, 29, 30, and 33 with 250 μg OVA i.n. Controls received phosphate-buffered saline (PBS, ThermoFisher Scientific) instead of OVA at the same time points (CN). Moreover, a SAA-group was treated with 4 mg/kg dexamethasone (Ratiopharm) i.p., 1 h before each challenge (SAA-DEX). For rechallenging experiments the mice were left for 19 days to recover from the acute inflammatory reaction before one re-exposure with 250 μg OVA i.n.

### XLF

In order to address lung function *in-vivo*, low dose planar cinematic x-ray images were acquired using an *in-vivo* microCT (QuantumFX, Perkin Elmer) operated with the following parameters: field of view 20 × 20 mm^2^, x-ray tube voltage 90 kVp and tube current 40 μA. 1024 images were acquired continuously with a rate of 30 images per second (Supplementary Movie 1). Mice were scanned unrestrained but under isoflurane anesthesia (~2% isoflurane in 2 l oxygen per min). The x-ray transmission over time in the lung area and in a background region were measured using Fiji^9^ and exported as text files. The developed quantification algorithm was implemented in Matlab R2011b (Mathworks).

### Determination of airway responsiveness to methacholine

For determination of responsiveness to methacholine by XLF, mice were anesthetized with isoflurane and placed in the microCT scanner as described under the previous point. An aerosol chamber (Aeroneb Pro, Inspiration Medical) was connected to the anesthesia tube via a T-fitting, so that the aerosolized methacholine was transported to the mouse with the isoflurane gas stream. XLF data were obtained at baseline and immediately following 10 sec nebulization of increasing concentrations of methacholine (0 mg/ml; 3 mg/ml; 10 mg/ml; 50 mg/ml), with 5 min intervals between each concentration.

### Unrestrained whole body plethysmography (UWBP)

Ventilation was measured by UWBP, measuring pressure changes resulting from the warming of the inspired air and cooling during expiration^10^. Mice were placed in a plexiglas chamber (1.2 L volume, custom made) that was connected to a differential low-pressure transducer (model DP1 03, Validyne Engineering, Northridge, CA). The second channel of the pressure transducer was connected to a reference chamber. The bias flow in the chamber was 200 ml/min, introduced by the suction of a CO_2_-sensor (Dräger). The pressure transducer was connected to a sine wave carrier demodulator (CD-15, Validyne Engineering). A MiniDigi1A (Molecular Devices) was used for digitization (1 kHz sampling rate) and storage on a PC Notebook (Fujitsu) using AxoScope Software (Molecular Devices). For analysis, pressure fluctuations were imported into LabChart 8 Software (ADInstruments) and offline band-pass filtered (0.5–20 Hz). The Peak Analysis Module was used to detect pressure maxima and calculate peak amplitude [a.u.], respiratory rate [**BPM**] and relative inspiratory time (**t**_**in**_**[%]**). **Max**- and **MinSlope** parameter, were derived as measure for chamber flow as the first derivative from pressure trace. Because animals were allowed to explore the chamber freely, and thus, some pressure changes resulted from sniffing behavior, we analyzed a period of 2 min after an initial period of adaptation (3 min).

### Use of experimental animals

All animal *in vivo* procedures were performed in compliance with the guidelines of the European Directive (2010/63/EU) and the German ethical laws and were approved by the administration of Lower Saxony, Germany (Nr. G15.1747).

### *In-situ* lung imaging using Synchrotron phase contrast micro CT (pSRμCT)

For detailed 3D depiction of asthma related anatomical alterations of the lung tissue, mice were sacrificed, lungs were kept *in-situ* and inflated with air at a constant pressure of 30 cm water column and the whole body was embedded in 1% agarose gel in cylindrical plastic containers as described before^11^. The samples were then imaged at the SYRMEP beamline (Synchrotron Light Source ‘Elettra’, Trieste, Italy) using the protocol described by Dullin *et al*.^11^. A sample-to-detector distance of 30 cm was chosen to allow for the formation of phase effects. Prior to 3D reconstruction a single distance phase retrieval algorithm (TIE-Hom, Gureyev T. *et al*.^12^) was applied resulting in the generation of 3D data sets with a voxel size of 9 × 9 × 9 μm^3^ predominately expressing the distribution of the δ-part of the complex refractive index within the sample (about 10 times more sensitive in lungs than using standard absorption based CT^13^). Quantification of the lung structure was performed by placing eight non-overlapping volumes-of-interests (VOIs) of 2 × 2 × 2 mm^3^ uniformly distributed in the peripheral region of the lungs in order to analyze the relative soft-tissue content of each VOI (**vol.ratio**) using threshold based segmentation to discriminate between air and non-air (soft-tissue and liquids). In addition, the average **δ-value** of the soft-tissue compartment was calculated. As the performed imaging cannot discriminate between liquid (such as mucus) and cells, the average **δ-value** is affected by the prevailing mixture of these components in any given mouse lung and can therefore be used as an additional parameter as demonstrated by Dullin *et al*.^6^. For 3D visualization and quantification the software Scry v6.0 (Kuchel & Sautter GbR) was used.

### Histology

Lungs were excised either after BAL or pSRμCT, fixed in 10% buffered formalin and embedded in paraffin. 3 μm-thick paraffin lung sections containing main stem bronchi were stained with either hematoxylin-eosin (H&E) for 2 min or with periodic acid for 5 min followed by Schiffs reagent for 15 min (PAS). An Axioskop 2 (Carl Zeiss Microscopy GmbH) microscope in combination with a Leica DC 100 camera was used for visualization of the stained sections. Sections were scored in a blinded fashion from 0 to 3 to address the amount of infiltrating immune cells seen in H&E (designated **HE-score**) and the amount of mucus producing goblet cells seen in the PAS staining (designated **PAS-score**), with 0 = no infiltrating cells and no mucus, 1 = low infiltrating cell count and mucus, 2 = medium infiltrating cell count and mucus, 3 = high infiltrating cell count and mucus.

### Bronchoalveolar Lavage (BAL)

Following lung function measurement 48 hrs after the last OVA challenge, mice were sacrificed. BAL was performed by washing the airways gently three times with 500 μl of 2% FCS/PBS after exposing and cannulating the trachea. Volumes were pooled and then washed once in the same buffer. Recovered cells were counted in a haemocytometer and 3 × 10^4^ cells were used for cytospins followed by Giemsa staining (Sigma Aldrich) for differential cell counting using an Axioskop 2 microscope. Values are expressed as total cell number and relative amount of eosinophils (**EOS[%]**).

### Statistical analysis

For statistical analysis, the unpaired Welch t-test for same mean implemented in MS Excel 2010 was used with a p-value of 0.1 (*) or a p-value of 0.05 (**) as margins for statistical significance.

### Note

*Ex-vivo* techniques such as histology and broncho-alveolar lavage provide only partial information in the analysis of allergic airway inflammation mouse models. While lung function measurements have been performed by plethysmography, this method shows limited sensitivity in allergic airway inflammation. Here we introduce a novel method to measure lung function in mice based on cinematic low dose planar x-ray imaging (XLF) using an *in-vivo* small animal microCT. We show that XLF is able to assess lung function in a severe allergic airway inflammation mouse model, provides comprehensive information to monitor treatment and is more sensitive than unrestrained whole body plethysmography. XLF can be easily adapted to other CT systems and other lung diseases and may therefore be of use to a broad audience.

## Additional Information

**How to cite this article:** Dullin, C. *et al*. X-Ray based Lung Function measurement–a sensitive technique to quantify lung function in allergic airway inflammation mouse models. *Sci. Rep*. **6**, 36297; doi: 10.1038/srep36297 (2016).

**Publisher’s note:** Springer Nature remains neutral with regard to jurisdictional claims in published maps and institutional affiliations.

## Supplementary Material

Supplementary Information

Supplementary Movie S1
