# Supplementary material for: X-Ray based Lung Function measurement–a sensitive technique to quantify lung function in allergic airway inflammation mouse models
Source: Sci Rep. 2016 Nov 2;6:36297. doi: 10.1038/srep36297 (PMC5090985; doi:10.1038/srep36297)
Supplement: Supplementary Information [file srep36297-s1.doc]

**X-Ray based Lung Function measurement – a sensitive technique to quantify lung function in allergic airway inflammation mouse models**

Authors: Dullin C^†a,b^, Markus MA^†c^, Larsson E^b^, Tromba G^b^, Hülsmann S^d^, and Alves F^a,c,e^

† equal contribution

1. Institute for Diagnostic and Interventional Radiology, University Medical Center Goettingen, Germany
2. Italian Synchrotron Light Source ‘Elettra’ Trieste, Italy
3. Max-Plank-Institute for Experimental Medicine, Dept. of Molecular Biology of Neuronal Signals, Goettingen, Germany
4. Clinic for Anesthesiology, UMG, Goettingen, Germany
5. Department of Hematology and Medical Oncology, UMG, Goettingen, Germany

Supplementary Figures:


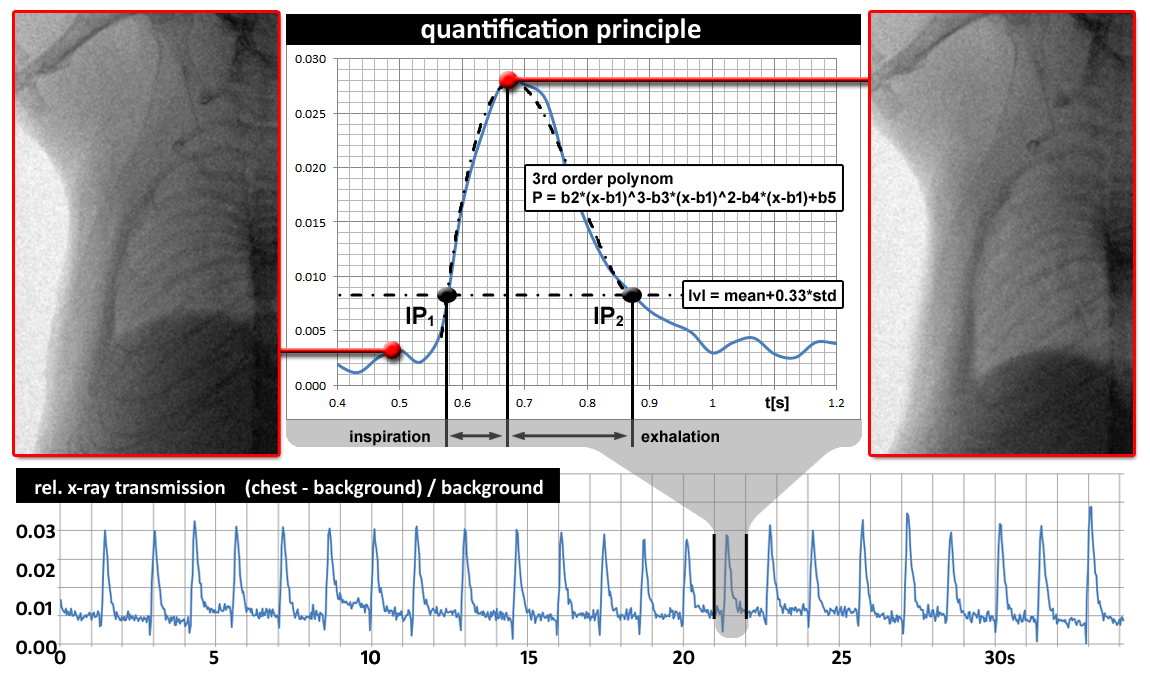


Supplementary Figure S1: Principle of the quantification of the XRT function. The change in x-ray transmission of the lung over 34 s is shown in the lower panel. Exemplified, one breathing peak in this XTF of a healthy mouse (CN) is shown in the upper middle panel. A level function (lvl) at mean+0.33*std is applied. A breathing peak is identified as the interval between two intersection points IP_1_ and IP_2_ of lvl and the XTF function. A third order polynom P is fitted into this interval. The percentage inspiration time t_in_[%] is calculated by inspiration time / (IP2-IP1). Quantification is repeated for all detected breathing peaks and the average of all parameter b_1_ to b_5_ and t_in_[%] is calculated. The left radiograph shows a frame at a time point in the expiration phase. The right radiograph is a frame at a time point in the inhalation phase.


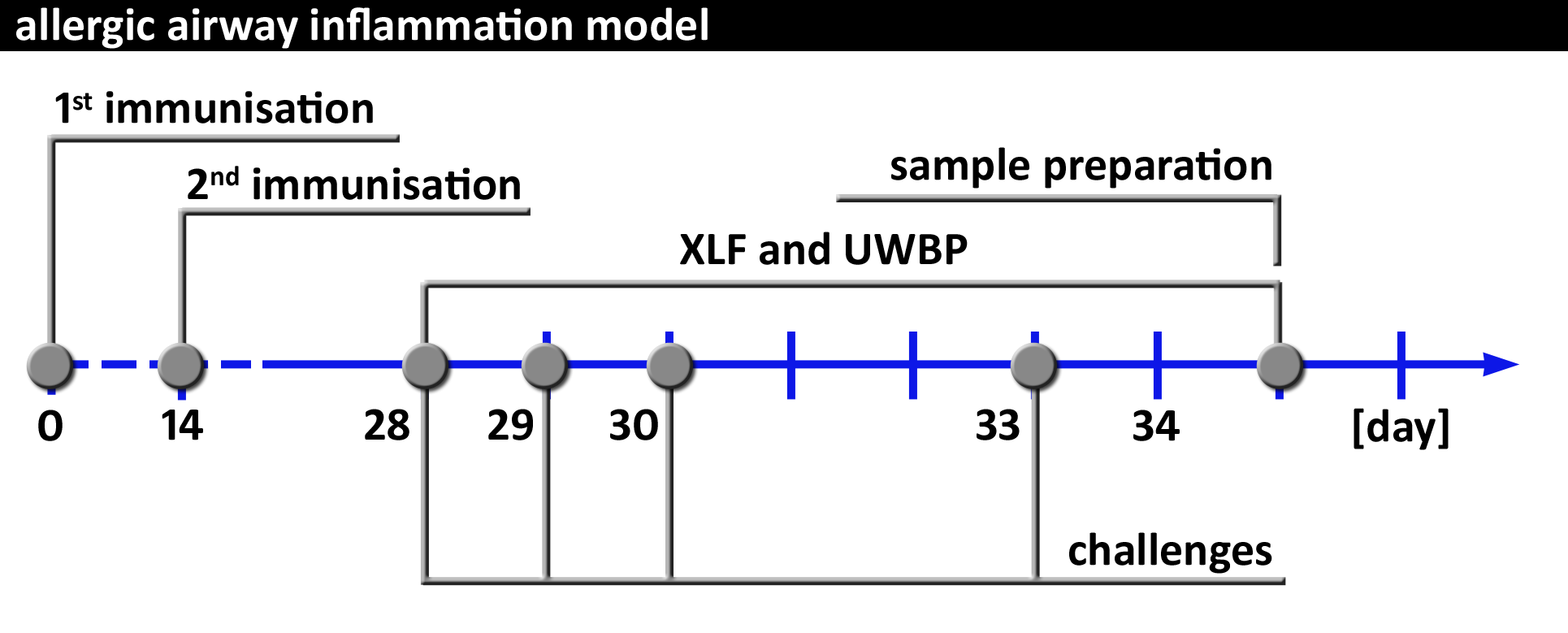


Supplementary Figure S2: Allergic airway inflammation model: mice were sensitized twice with 50µg OVA i.p. and i.n. on days 0 and 14 and then challenged 4 times on days 28, 29, 30, and 33 with 250µg OVA i.n. (SAA). Controls received PBS instead of OVA at the same time points (CN). Moreover, a SAA-group was treated with 4 mg/kg dexamethasone i.p., 1 h before each challenge (SAA-DEX). XLF and UWBP were performed shortly before the 1^st^ OVA challenge and 2 days after the last challenge. Following the last XLF, mice were either processed for BAL or prepared for pSRµCT analysis. Following the pSRµCT measurement, lungs were explanted and processed for histology.


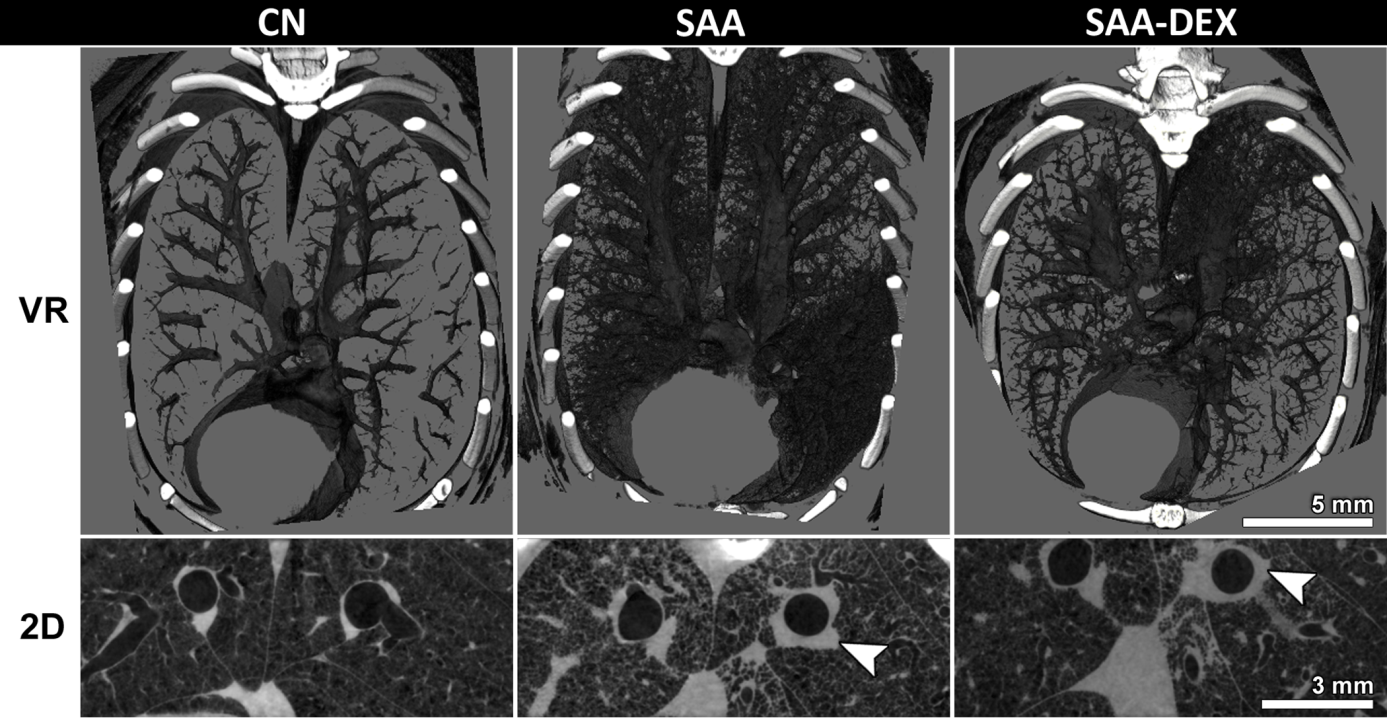


Supplementary Figure S3: Structural analysis of asthma hallmarks by pSRµCT. Representative volume renderings (VR) of 2 mm virtual cuts of lungs are shown in the upper panel. Clearly a higher soft-tissue volume ratio can be found in lungs of SAA than in the control CN mice (upper panel). The treated mouse, SAA-DEX, shows reduced soft-tissue content meaning a reduction of the swelling of the airways compared to SAA, but still higher than the control CN. The same results can be seen in 2D (lower panel). The strong swelling of the bronchial walls in a SAA mouse (middle panel) is indicated by a white arrow head. The lung of a SAA-DEX mouse shows a reduction in the parameter soft-tissue vol.ratio, however a swelling of the airway walls is still visible (white arrow head).


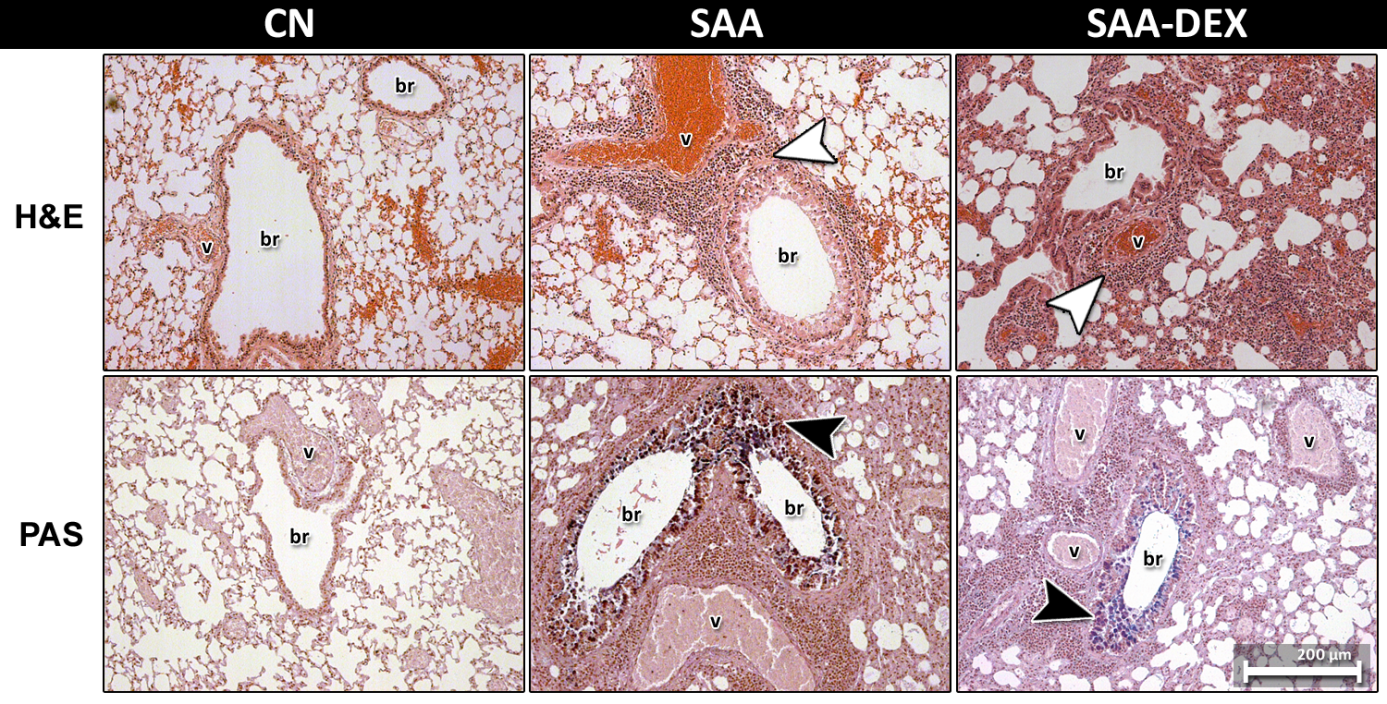


Supplementary Figure S4: Histological analysis of lungs of the same mice shown in Supplemental Figure S3. Upper row shows paraffin lung tissue sections stained with H&E in which infiltrating immune cells can be identified by their dark stained nucleus (white arrow heads) in SAA and SAA-DEX samples. The SAA mouse (middle panel) demonstrates a large amount of infiltrating cells between a blood vessel and a bronchus. Compared to the control CN (left panel), lung tissue of SAA-DEX shows still more infiltrating cells indicating inefficient dexamethasone treatment. In the lower row PAS staining of corresponding lung sections are presented that highlights mucus producing cells in purple (black arrow heads). Strong mucus production can be seen in lungs of SAA (purple staining, middle panel) and to a slightly lower extent in SAA-DEX mice (right panel). Lungs of CN mice (left panel) show no mucus production. (br = bronchi, v = blood vessel).


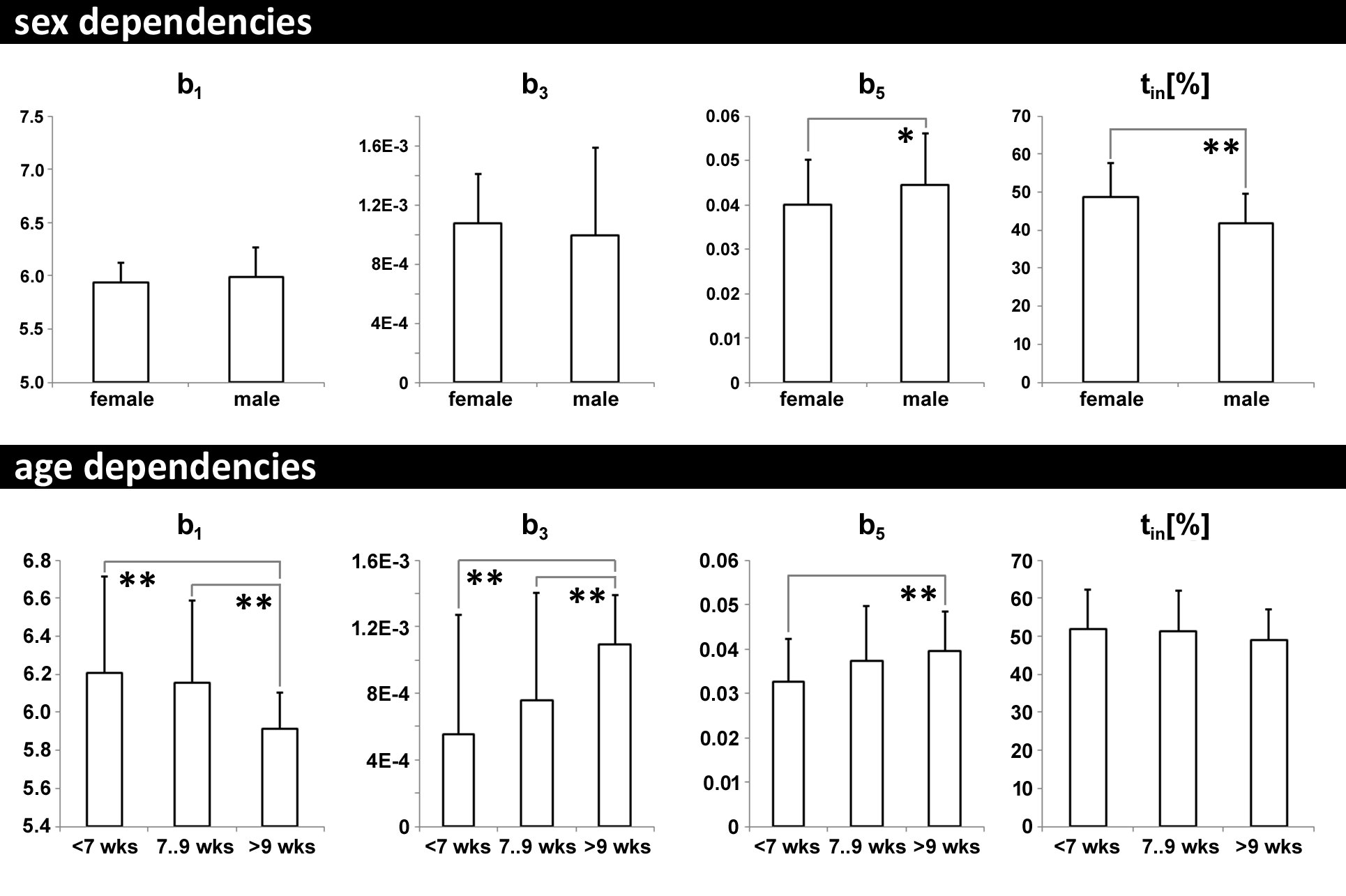


Supplementary Figure S5: Influences of age and sex on parameters of XLF in healthy mice. In order to analyse sex dependencies (upper panel), female (N=37) and male mice (N=32) older than 7 weeks were analysed. Only the parameter b_5_ and t_in_[%] show significant differences, most likely due to the larger body size of the males. For age dependency analysis (lower panel), healthy female mice were grouped into 3 groups, younger than 7 weeks (N=16), between 7 and 9 weeks (N=27) and older than 9 weeks (N=29). Except t_in_[%] all parameters show significant age related variations. Statistical significance difference of the results is indicated by (* p<0.1, ** p<0.05).

Supplementary Movie 1 Principle of the XLF method. The movie shows how the x-ray transmission function is composed by measuring the normalized x-ray attenuation over the mouse chest. Two breathing events are shown of a control and an asthmatic mouse.
